# Body Composition’s Association with Resting Energy Expenditure Prediction in a Large Population Sample from Different Age Groups, Sex, and Physical Activity Levels

**DOI:** 10.3390/jfmk11010101

**Published:** 2026-02-27

**Authors:** Lucas Bertoluci Zuquieri, Gabriel de Souza Zanini, Danilo Alexandre Massini, Eliane Aparecida de Castro, Wellington Segheto, Cassiano Merussi Neiva, Pedro José Benito, Dalton Müller Pessôa Filho

**Affiliations:** 1 Department of Physical Education, School of Sciences (FC), São Paulo State University (UNESP), Bauru 17033-360, Brazil; lucas.b.zuquieri@unesp.br (L.B.Z.); gabriel.zanini@unesp.br (G.d.S.Z.); dmassini@hotmail.com (D.A.M.); merussi.neiva@unesp.br (C.M.N.); 2 Postgraduate Program in Movement Sciences, São Paulo State University (UNESP), Bauru 17033-360, Brazil; 3 Postgraduate Program in Human Development and Technology, São Paulo State University (UNESP), Rio Claro 13506-900, Brazil; elianeaparecidacastro@gmail.com; 4 Instituto Federal de Educação, Ciência e Tecnologia do Espírito Santo (IFES), Vitória 29056-264, Brazil; 5 Instituto Federal de Educação, Ciência e Tecnologia do Sudeste de Minas Gerais, Barbacena 36205-018, Brazil; wsegheto@gmail.com; 6 LFE Research Group, Universidad Politécnica de Madrid (UPM), 28040 Madrid, Spain; pedroj.benito@upm.es

**Keywords:** resting energy expenditure, predictive equations, body composition, dual X-ray absorptiometry, adults, older, sex, physical activity level

## Abstract

**Background:** Resting energy expenditure (REE) represents 60–75% of total daily energy expenditure and is mainly determined by fat-free mass (FFM). Indeed, the predictive equations vary according to FFM techniques and population characteristics. Therefore, this study aimed to explore the influence of dual-energy X-ray absorptiometry (DXA)-derived FFM on REE prediction by different predictive equations in a large and diverse cohort. **Methods:** A total of 1987 active and sedentary participants of both sexes (43.8 ± 19.4 years) underwent body composition assessment by DXA. REE was predicted using the Harris–Benedict, Schofield, Mifflin–St Jeor (weight- and height-based), and Mifflin (FFM-based) equations. Statistical analyses included Kruskal–Wallis, Spearman correlations, and linear regression. **Results:** Men presented higher absolute FFM, whereas women exhibited higher relative fat mass (FM) (*p* < 0.01). Across age groups, FFM declined progressively, while FM increased (*p* < 0.01). The REE differed significantly (*p* < 0.001) between equations, with the lowest values predicted from the FFM-based model, while the Harris–Benedict and Schofield equations showed the highest REE, especially in women. Strong correlations were observed between FFM and REE (r = 0.77–0.98; *p* < 0.01) for all age groups and equations, whereas FM showed strong correlations (r = 0.77–0.85; *p* < 0.01) only for the ≥60 years group. REE tended to be higher in active than sedentary participants, with the correlations to FFM and FM exhibiting a similar profile to that observed for the whole group. **Conclusions:** FFM showed a strong association with the estimate of REE in active and sedentary participants from both sexes and different age groups, but FM showed a similar trend in older participants only. Therefore, the increase or the maintenance of FFM with an active lifestyle is important to keep REE at high and efficient levels regardless of sex and age.

## 1. Introduction

Resting energy expenditure (REE) corresponds to the minimum amount of energy required to sustain vital physiological functions in a post-absorptive resting state and accounts for approximately 60–75% of total energy expenditure in individuals with a sedentary lifestyle [1,2]. Although the terms REE and basal metabolic rate (BMR) are often used interchangeably, they differ in methodological aspects: BMR requires highly standardized conditions, whereas REE is more applicable in both clinical and research contexts [1,3].

Interindividual variability in REE is primarily determined by fat-free mass (FFM), which is recognized as the strongest predictor of this component [4,5]. However, the contribution of FFM is not uniform, as REE results from the sum of the specific metabolic rates of different organs and tissues. Structures such as the liver, brain, kidneys, and heart, although representing less than 6% of total body mass, account for more than 50% of REE, whereas skeletal muscle, despite its larger mass, exhibits a lower specific metabolic rate [2]. In addition, factors such as age, sex, height, body composition, and ethnicity significantly modulate REE [4,6].

In clinical and research contexts, direct measurement of REE using indirect calorimetry (IC) is considered the gold standard [7,8,9,10]. IC relies on the measurement of oxygen consumption and carbon dioxide production, followed by application of the Weir equation to calculate REE [7,9]. However, logistical and financial limitations often favor the routine use of predictive equations [11,12,13]. Despite their widespread application, recent evidence demonstrates that these equations frequently increase the variation of REE estimates in specific populations, such as women with excess body fat [3] or older adults [6], thus compromising the accuracy of nutritional recommendations and weight management strategies.

In this context, highly accurate techniques such as dual-energy X-ray absorptiometry (DXA) allow for a detailed assessment of body composition and enable investigation of how FFM influences REE estimates across diverse population groups, including the physical activity level. Studies conducted in different countries suggest that models based on FFM are more robust than those relying solely on weight and height [14,15,16,17]. Nonetheless, there is a scarcity of studies conducted with a large and diverse cohort of populations using DXA, which represents a relevant gap in the literature. Furthermore, previous studies in adults have demonstrated that absolute REE (kcal/day) declines with advancing age [6,18,19,20]. However, although this phenomenon is well established, it remains unclear whether these changes are associated with alterations in body composition or with metabolic activity itself, particularly across different populations and socioeconomic contexts [21]. An increase in fat mass (FM) content and a sedentary lifestyle have been considered factors influencing REE reduction with aging [2,5].

Beyond constitutional and demographic determinants, the maintenance of FFM across the lifespan is strongly influenced by habitual physical activity levels and long-term exposure to mechanical and metabolic stimuli derived from exercise. Recent evidence indicates that physically active individuals and athletes consistently present higher absolute and relative FFM, improved muscle quality, and greater mitochondrial density when compared with sedentary peers, resulting in higher and more metabolically efficient resting energy expenditure profiles. From a functional perspective, exercise-induced adaptations in skeletal muscle, such as increased oxidative capacity, improved neuromuscular efficiency, and enhanced muscle protein turnover, contribute not only to movement performance but also to the preservation of resting metabolic demand, particularly across aging and in clinical populations [22,23,24].

Importantly, the type of exercise plays a decisive role in modulating the FFM–REE relationship. Resistance training and multimodal exercise programs have been consistently shown to attenuate age-related declines in lean mass, preserve functional capacity, and sustain resting energy expenditure by maintaining metabolically active tissue [25,26,27]. In contrast, sedentary behavior accelerates FFM loss and favors disproportionate fat mass accumulation, which may increase REE through low-grade inflammation and compensatory metabolic costs rather than through efficient oxidative metabolism [28,29,30]. Thus, from a kinesiology and functional morphology standpoint, preserving FFM via regular exercise represents a central mechanism linking movement, metabolic health, and energy balance regulation.

Therefore, the present study aimed to examine the influence of FFM and FM, assessed by DXA, on REE estimated by different predictive equations in a large sample of participants of both sexes, different age groups and physical activity levels, while discussing implications for clinical practice and healthy lifestyle. The hypothesis was that the wide variability in age, sex, and physical activity levels within the population cohort significantly modulates REE prediction. Moreover, applying a diverse range of predictive equations was expected to demonstrate varying levels of correlation to FFM and FM across these demographic strata, thereby identifying the sensibility of each model to body composition alteration in this specific population. In addition, while FFM remains the primary driver of REE due to the metabolic scaling of vital organs, the strength of this relationship varies according to physical activity status; this status counteracts age-related muscle decline, whereas the accumulation of FM in sedentary individuals strengthens its correlation to REE.

## 2. Materials and Methods

**Sample**

Data were obtained from 846 men and 1141 women who underwent DXA scans between 2012 and 2019 at the Laboratory for Human Sports Performance Optimization (LABOREH). The sample had a mean age of 43.8 ± 19.4 years and comprised individuals with a wide range of anthropometric and functional profiles, including sedentary and physically active participants and those with normal weight, overweight, or obesity, as well as diverse socioeconomic strata and ethnic–racial backgrounds. The following specific exclusion criteria were applied: (1) individuals under 20 or over 79 years of age; (2) ethnic groups with insufficient representation (*n* < 20) when stratified by age and sex; (3) participants with physical or mental disabilities that could compromise the DXA scan; (4) individuals with debilitating chronic comorbidities, active cancer, or those in outpatient/clinical settings; (5) athletes undergoing rehabilitation for injuries affecting muscle mass; and (6) self-reported use of ergogenic aids or recent limb immobilization. These exclusion criteria were applied to mitigate potential sources of bias and reduce noise stemming from clinical conditions that directly influence body composition independent of age and physical activity level. Participants self-reported their weekly physical activity levels during the screening for body composition assessment. The International Physical Activity Questionnaire (IPAQ)—short form (last 7 days, self-administered) was utilized [31]. Based on the World Health Organization (WHO) guidelines, individuals were classified as active if they performed at least 150 min of moderate-intensity aerobic physical activity or 75 min of vigorous-intensity activity per week. Those who did not meet these criteria were classified as sedentary [32].

The dataset was consolidated from multiple research projects conducted during the study period, thereby ensuring heterogeneity and representativeness across body composition and demographic characteristics. This diversity strengthened the analysis of the relationship between body composition and REE, as it encompassed a broader spectrum of interindividual variability and reduced potential biases associated with restricted subgroups. This research was approved by the Ethics Committee of the University under the registration CAAE: 70076317.1.0000.5398; informed consent was obtained from all subjects involved in this study. Written informed consent has also been obtained from the participants for the publication of this paper.

**Anthropometric and Body Composition Profile**

Anthropometric measures of body mass and height were obtained using a calibrated scale and stadiometer (W200A, Welmy^®^, Santa Bárbara d’Oeste, Brazil), with a precision of 0.1 kg and 0.1 cm, respectively. During the assessments, participants wore light clothing and were barefoot, following international recommendations for standardized anthropometric measurement.

Body mass index (BMI) was calculated as body mass divided by height squared (kg/m^2^). For classification purposes, two criteria were adopted: (i) the World Health Organization cut-off points for adults (BMI < 18.5 kg/m^2^ = underweight; 18.5–24.9 kg/m^2^ = normal weight; 25–29.9 kg/m^2^ = overweight; ≥30 kg/m^2^ = obesity) and (ii) the Lipschitz criteria for older adults (≥60 years), in which BMI < 22 kg/m^2^ = underweight; 22–27 kg/m^2^ = normal weight; >27 kg/m^2^ = overweight [33,34].

Body composition was assessed by DXA (QDR Discovery Wi^®^ Hologic^®^, Danbury, CT, USA), according to validated protocols described in the literature [17,35]. Absolute and relative values of fat mass (FM), FFM, and total body mass were obtained. All scans were processed using Hologic APEX^®^ software (v. 3.3), ensuring consistency and comparability of results. The use of DXA as the reference method allowed for precise quantification of body compartments, minimizing the limitations of indirect approaches and enabling more robust analyses of the association between FFM and REE.

**Predictive Equations for Resting Energy Expenditure (REE)**

REE was estimated using different predictive equations widely applied in clinical practice and population-based research. Three classical FFM non-based equations were selected, as they are internationally recognized and recommended in nutritional and metabolic assessment guidelines. (i) Harris and Benedict [11]: developed based on body mass, height, age, and sex and historically one of the most applied equations; (ii) Schofield [13]: derived from a large database, this equation uses body mass as the primary predictor, with adjustments for different age groups; and (iii) Mifflin et al. [12]: a more recent equation that includes body mass, height, age, and sex, considered the most appropriate for use in populations with overweight and obesity. In addition, a fourth equation in which FFM value is the unique estimator was also selected to analyze the influence of body composition across different age groups. This multi-model approach was essential to identify how sex, age, and physical activity levels influence the prediction of REE values within the large and diverse cohort of Brazilian participants. The respective equations are presented in Table 1.

**Statistical Analysis**

Statistical analysis initially included descriptive statistics, expressed as means and standard deviations for all continuous variables. Normality of distributions was assessed using the Kolmogorov-Smirnov test, which did not confirm normality for the main variables. Consequently, nonparametric procedures were adopted. Comparisons of FM and FFM across different age groups were performed using the Kruskal-Wallis test, followed by post hoc analyses with the Mann-Whitney test and Bonferroni correction. Comparisons among REE predictive equations were conducted using the Friedman test, with post hoc analyses performed using the Wilcoxon test and Bonferroni adjustment. Sex-based comparisons for each equation were carried out using the Mann-Whitney test.

Linear correlations between variables were assessed using Spearman’s rank correlation coefficient and classified according to very strong (≥0.90), strong (0.70–0.89), moderate (0.50–0.69), weak (0.30–0.49), and very weak (<0.30) [36]. In addition, multiple linear regression analyses were performed to examine the relationship between FM, FFM, and REE values estimated by predictive equations. Cases in which residuals did not meet the assumption of normality were excluded to ensure model validity. Outliers were identified and removed based on a standardized residual threshold of ±3 standard deviations. Linearity and homoscedasticity were assessed via scatterplots of standardized residuals against standardized predicted values, while the normality of the error distribution was confirmed using normal P-P plots (i.e., the cumulative probability of the data against the theoretical cumulative probability of a normal distribution). All analyses were conducted using SPSS software (version 20.0, IBM Corp., Armonk, NY, USA), and graphs were generated with GraphPad Prism (version 8.0, GraphPad Software, San Diego, CA, USA). The significance level was set at *p* ≤ 0.05, with a 95% confidence interval.

## 3. Results

**Figure 1:**
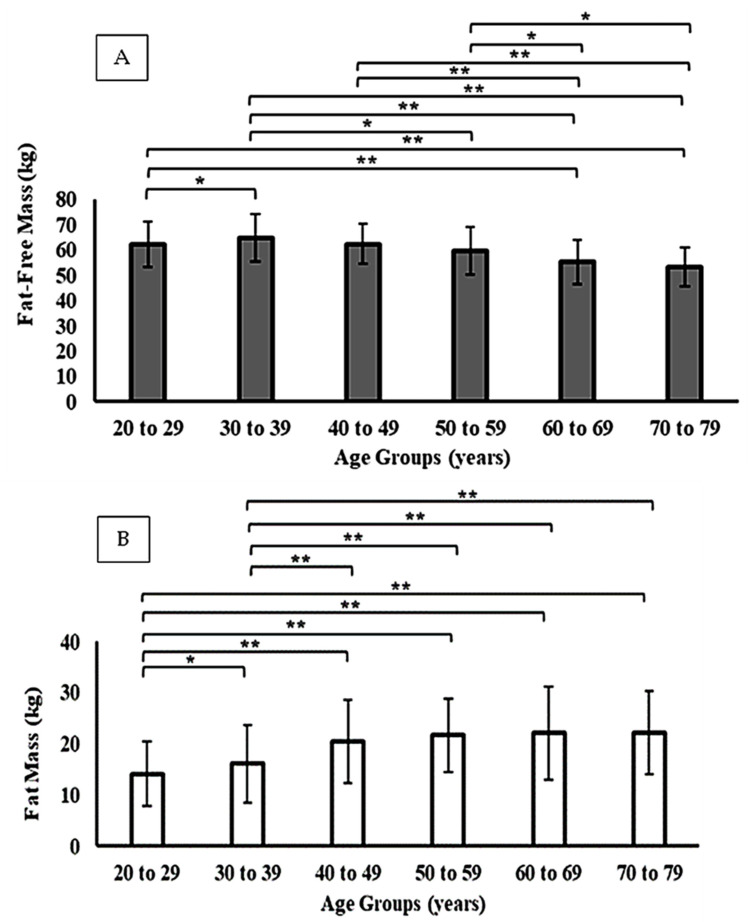
Fat-free mass (Panel (**A**)) and fat mass (Panel (**B**)) across different age groups in men. * *p* < 0.05; ** *p* < 0.001.

**Figure 2:**
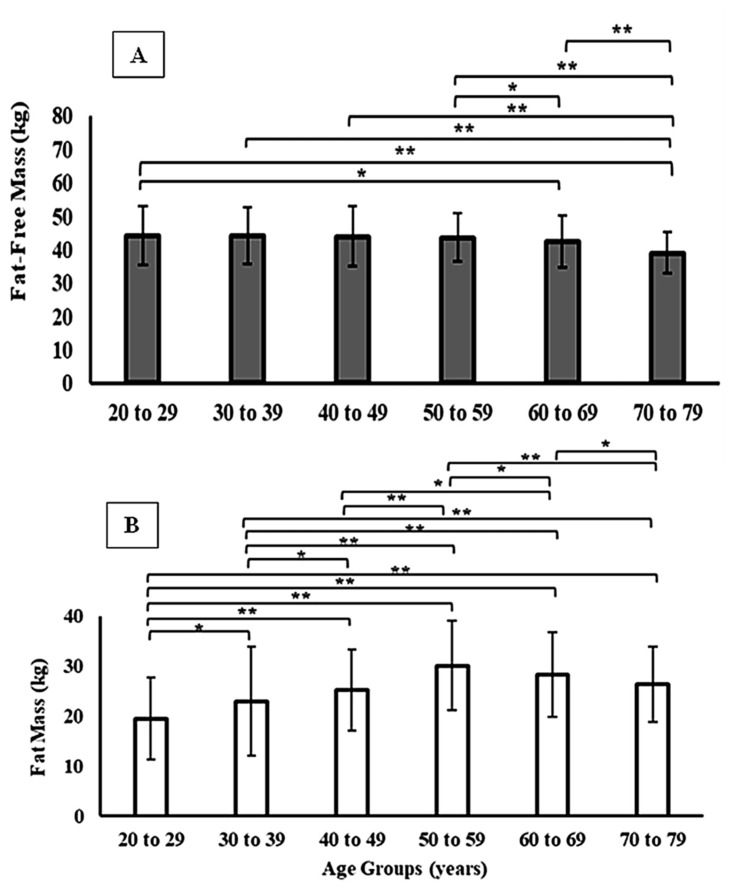
Fat-free mass (Panel (**A**)) and fat mass (Panel (**B**)) across different age groups in women. * *p* < 0.05; ** *p* < 0.001.

When stratified by sex, men exhibited consistently higher absolute values of FFM across all age groups, while women presented higher relative FM values (*p* < 0.01). In both sexes, FFM declined progressively from the fifth decade of life, accompanied by an increase in FM (Figure 1 and Figure 2). However, the magnitude of these changes differed by sex: men showed a steeper decline in FFM with aging, whereas women displayed a more pronounced increase in relative FM, particularly after 50 years. These sex-specific differences are detailed in Table 2. This tendency is also observed when comparing FFM and FM between participants grouped according to their level of physical activity (i.e., active vs. sedentary). For active men and women, FFM and FM showed higher and lower values, respectively, compared to their sedentary peers among both younger (≤29 years) and middle-aged adults (30–59 years). However, these differences were only statistically significant among men (Table 3).

Comparison among REE predictive equations (Table 4) revealed consistent discrepancies. The Friedman test indicated significant differences in estimated values (*p* < 0.01), with Wilcoxon post hoc analysis showing that the FFM-based Mifflin equation showed the lowest values of REE. In contrast, the Harris–Benedict and Schofield equations tended to show higher REE values than Equations (3) and (4), particularly in women regardless of the age group. Conversely, the body mass–based Mifflin model predicted lower REE values in individuals with overweight and obesity. Sex-based comparisons conducted using the Mann–Whitney test indicated that men had significantly higher estimated REE values across all equations and age groups (*p* < 0.01). However, when adjusted for FFM, these differences were attenuated, suggesting that sex-related discrepancies are largely attributable to greater amounts of metabolically active mass in men. Similarly, when comparing REE estimates between active and sedentary participants, men exhibited higher values than women in both groups (*p* < 0.01), regardless of age group (Table 5). Additionally, Table 5 indicates an overall tendency for REE estimates to differ between active and sedentary participants (*p* < 0.05); however, when analyzed by sex and age group, this difference was confirmed only for men in the younger group (≤29 years; *p* < 0.01). Moreover, REE estimates differed significantly across age groups, with values decreasing as age increased (*p* < 0.01) for both sexes and all physical activity levels.

Correlation analyses (Table 6) demonstrated that FFM was strongly associated with REE values across all equations (r ranging from 0.70 to 0.85; *p* < 0.01), whereas FM showed weak to moderate correlations (r ranging from 0.30 to 0.55; *p* < 0.05). Exceptionally for the age group over 60 years, the FM considerably improved the coefficient of correlation in both sexes (r ranging from 0.47 to 0.89; *p* < 0.01), regardless of the equation. According to the adopted classification, the strength of the correlation between FFM and REE was considered strong for most equations and age groups, while the contribution of FM was strong only for the ≥60 years group. A similar correlational tendency is observed when participants are grouped according to their physical activity level. Table 7 shows strong correlations between REE estimates and FFM, regardless of physical activity level for both sexes. Furthermore, an increased correlation level between REE estimates and FM was noted as age increased. Finally, linear regression models confirmed the strong influence of FFM on REE in both sexes, regardless of the predictive equation applied. Cases in which residuals did not meet the assumption of normality were excluded to ensure the robustness of the statistical models (Table 8).

## 4. Discussion

The present study provided a comprehensive overview of how different predictive equations to estimate REE perform across various demographic and physical activity profiles and also investigated the relationship between FFM, FM, and REE based on measurements obtained by DXA in a large and diverse cohort of Brazilian participants. The findings reinforce the notion that FFM is the primary determinant of REE, supporting evidence that up to 80% of the interindividual variability in resting metabolism can be explained by the amount of metabolically active tissue [4,5,22,37]. Although FM also contributes to energy expenditure, its role is modest, consistent with classical findings by Nelson et al. [5], which demonstrated a secondary contribution of FM once adjusted for FFM.

The results further confirm that the relationship between REE and FFM is not homogeneous but rather modulated by constitutional and functional factors. Studies by Wang et al. [1,17] indicate that the linear association between REE and FFM in adults conceals greater complexity, since different organs and tissues exhibit highly distinct specific metabolic rates. Organs such as the liver, heart, kidneys, and brain, which represent only 5–6% of total body mass, may account for more than 50% of REE [2,38]. Magnetic resonance imaging and IC studies show that small variations in organ mass can explain substantial interindividual differences in REE, surpassing the influence of age, sex, or race [39,40]. This imbalance helps explain why equations based solely on FFM or body mass present limitations when applied to heterogeneous populations.

From a kinesiology and exercise physiology perspective, the strong association observed between FFM and REE in the present study highlights the functional relevance of lean mass as a metabolically active tissue shaped by chronic exposure to mechanical loading and neuromuscular activation. Fat-free mass is not a static compartment but rather a highly plastic tissue whose quantity and quality are strongly influenced by physical activity level, exercise modality, and training history [41,42]. Evidence from physically active individuals and athletes demonstrates that regular engagement in resistance and multimodal exercise promotes the preservation of lean tissue, enhances muscle oxidative capacity, and sustains higher resting energy expenditure when compared to sedentary counterparts, even when total body mass is similar [43,44,45].

In contrast, sedentary behavior accelerates the decline of FFM and favors disproportionate fat mass accumulation, leading to alterations in the physiological determinants of REE. Although FM is traditionally considered a low-metabolic-rate tissue, excess adiposity is associated with chronic low-grade inflammation, increased sympathetic activation, and compensatory metabolic demands that may elevate resting energy expenditure through inefficient and potentially pathological pathways [19,46,47,48]. This mechanism provides a plausible explanation for the stronger associations observed between FM and REE in older age groups, as identified in the present study, reflecting a shift from an efficient, lean mass–driven metabolism toward a metabolically costly and less functional body composition profile [19,20,49,50].

Regarding age, we observed a trend toward lower absolute REE in older individuals, even after adjustment for FFM. This finding is consistent with Fukagawa et al. [6], who reported reductions in resting metabolic rate among older adults not fully explained by lean mass loss but also by qualitative alterations in tissue metabolic activity. These data support the hypothesis that aging leads not only to quantitative declines in FFM but also to reduced cellular metabolic efficiency, possibly associated with mitochondrial and enzymatic changes [2,18,24,51].

Importantly, exercise training appears to modulate both quantitative and qualitative aspects of FFM during aging. Longitudinal studies demonstrate that resistance training and combined exercise programs attenuate sarcopenia, preserve muscle fiber cross-sectional area, and improve mitochondrial function, thereby mitigating the age-related decline in REE. In this context, the maintenance of FFM through exercise represents not only a strategy to preserve movement capacity and functional independence but also a key mechanism for sustaining resting metabolic demand and metabolic health across the lifespan [52,53,54].

Sex-related differences were also evident, with men presenting higher absolute values of FFM and REE, corroborating previous studies highlighting physiological differences driven by greater muscle mass and distinct organ and tissue distribution between sexes [4,23,55,56]. However, when REE was adjusted for FFM, these differences were attenuated, suggesting that the critical determinant lies in the relative amount of metabolically active tissue rather than biological sex per se [23,55].

Recent evidence from athlete populations reinforces this interpretation, showing that when matched for FFM and training status, sex differences in REE are substantially reduced or eliminated. This finding underscores the relevance of functional morphology and training-induced adaptations over biological sex alone in determining resting metabolic demand, aligning with a movement-centered and performance-oriented interpretation of energy metabolism [23,29,55].

The selection of the predictive equations for the current analysis was based on their widespread international recognition and their distinct predictive characteristics, which allowed for a comprehensive analysis of REE in a large cohort of the population. For example: Equation (1) (Harris–Benedict, 1918) [11] was chosen as a historical and widely applied benchmark in clinical practice, utilizing body mass, height, age, and sex as predictors; Equation (2) (Schofield, 1985) [13] was included because it is derived from a large database and specifically adjusts its calculations for different age groups, primarily using body mass as the predictive variable; Equation (3) (Mifflin et al. 1990—weight-based) [12] was selected for its reported superior accuracy in populations with overweight and obesity, providing a modern comparison to the classical Harris–Benedict model; and Equation (4) (Mifflin et al. 1990—FFM-based) [12] was specifically chosen to isolate the influence of body composition, since the use of fat-free mass (FFM) as the unique estimator by this equation has allowed for exploring how metabolically active tissue modulates REE independent of total body weight.

The comparative analysis of these classical predictive equations indicates that the Harris–Benedict, Schofield, and Mifflin models showed a propensity to yield higher REE estimates in specific population groups. For instance, the Harris–Benedict and Schofield equations consistently resulted in higher predicted REE in sedentary populations or those with lower metabolic rates, such as the elderly [1,57]. Conversely, the Mifflin equation is widely recognized for its higher accuracy (often exceeding 70–80% in specific BMI strata) in individuals who are nonobese and with overweight or obesity [58,59]. Furthermore, models based solely on FFM (such as the Mifflin-FFM) tend to be more precise for physically active individuals or athletes, as they account for the higher metabolic cost of lean tissue [5,60]. In addition, Maury-Sintjago et al. [3] demonstrated that the Harris–Benedict, Schofield, and Mifflin equations tended to provide higher values when estimating REE in young women with excess adiposity, a finding consistent with our results and reinforcing the need for caution when applying generalized equations in clinical settings, particularly among populations with body composition profiles different from those used to derive the original models. Other studies have also reported a systematic higher REE estimate by the Harris–Benedict, Schofield, and Mifflin equations when compared to measurements obtained via indirect calorimetry in athletes, obese and non-obese individuals, and healthy older adults [60,61]. However, REE estimates from the Harris–Benedict and Mifflin equations have provided values with high accuracy compared to other predictive models and are in agreement with equations based on FFM estimators [5,60]. In fact, the Mifflin-FFM estimate of REE in the current study yielded values close to the reference REE for young men (≤30 years and 18% FM). This reference is based on the oxygen uptake rate for muscle and organs (~6946 kJ/24 h or ~1658 kcal/24 h), as recommended by Nelson et al. [5].

Hence, by including this variety of models, the current study aimed to identify which predictive approach aligns most closely with the physiological characteristics of the population across different life stages. Taking into account the stronger association of REE estimates with FFM (independent of sex, age, and physical activity level), the Mifflin-FFM displays superior alignment for analyzing REE in this cohort, despite the lack of validation against a gold-standard method (i.e., indirect calorimetry). However, given the increase in FM with age and sedentary behavior, the Harris–Benedict and Schofield equations remain relevant for estimating REE in these specific contexts. From this perspective, direct body composition assessment using DXA or equivalent methods offers greater consistency in estimating REE, especially for nutritional prescription and metabolic monitoring [62].

From an applied exercise and training perspective, inconsistent REE estimates may directly compromise nutritional planning, recovery strategies, and body composition management in both physically active individuals and clinical populations. The systematic tendency to produce higher REE values may lead to excessive caloric intake and fat mass gain, whereas estimates resulting in lower predicted REE may impair recovery, adaptation, and lean mass maintenance. Therefore, incorporating direct assessments of FFM, particularly via DXA, provides a more consistent framework for exercise prescription, nutritional periodization, and long-term metabolic health interventions.

Finally, the current findings contribute to the growing body of evidence demonstrating that while FFM remains a primary driver of REE, its predictive dominance is modulated by age and physical activity. It is important to emphasize that this study did not intend to validate these equations or determine a single superior model; rather, it aimed to analyze how different predictive approaches behave across a highly diverse population cohort. The results also reveal that as aging and sedentary behavior progress, the expansion of adipose tissue (FM) begins to exert a more pronounced influence on metabolic estimates, suggesting that traditional anthropometric models may implicitly capture this shifting metabolic reality in specific strata. Selecting a predictive model should, therefore, be guided by the body composition and lifestyle profile of the population.

Despite the strengths of this study, including its large and heterogeneous sample spanning different ages, sexes, and anthropometric profiles, combined with the use of DXA, several limitations must be acknowledged. The absence of direct measurement by IC, considered the gold standard, restricted evaluation of the absolute accuracy of predictive equations. Consequently, the current analysis focused on the comparative performance of established predictive models rather than absolute validation. Despite this limitation, the equations presented in Table 1 were strategically selected for their specific strengths and widespread clinical use. Furthermore, as these predictive equations were originally developed in populations distinct from the current sample of participants’ context, systematic bias may have been introduced into the estimates. Therefore, we acknowledge the need for future studies involving direct metabolic measurements to identify which predictive approach aligns most closely with the characteristics of the population across different life stages. The cross-sectional design precluded inferences regarding intraindividual changes over time. In addition, although our cohort represents a wide range of ages and body compositions, its origin from a single region and the predominance of a specific ethnic–racial profile may limit the generalizability of the findings to the full ethnic diversity of the Latin American population. However, the cohort consisted of 1987 individuals (846 men, 1141 women) who voluntarily sought body composition assessment; thus, it encompasses a wide range of body compositions and age groups, which are the primary focus of this predictive equation analysis. Moreover, the current study did not account for FM and organ-specific mass, which are key physiological determinants of energy expenditure. While our primary objective was to categorize participants based on adherence to international health-promoting standards versus sedentary risk, we acknowledge that the absence of intermediate activity data and specific training modalities is a limitation for inferring the specific potential of each equation across the diverse activity profiles of the participants.

Future investigations integrating indirect calorimetry, longitudinal exercise interventions, and detailed assessments of muscle quality and functional performance (e.g., physical activity duration, intensity levels, and multiple domains—occupational, transport, leisure, and sports) may further elucidate how training-induced adaptations in FFM influence resting energy expenditure across different populations. Such approaches would strengthen the translational relevance of REE research within the fields of kinesiology, exercise physiology, and functional morphology.

## 5. Conclusions

The results reinforce that FFM exerts a strong influence on REE estimates; consequently, equations that do not account for FFM showed a propensity to yield higher energy requirement values in relevant subgroups. Therefore, equations based on DXA-derived FFM measurements demonstrated a tendency to maintain more consistent REE predictions independent of sex, age, and physical activity level, emerging as a robust framework for both clinical and research settings. The development of equations calibrated for local populations, validated against indirect calorimetry, and ideally informed by organ–tissue modeling, represents the next step toward reducing prediction discrepancies and enhancing individualized clinical prescription.

Furthermore, the findings highlight the importance of considering sex, age, and body composition as modulators of REE prediction. Our data showed that the predicted values of REE tended to be higher in active participants compared to sedentary ones, particularly in young men. Evidence indicated that the prediction of REE is significantly lower in older individuals when expressed in absolute values (kcal/day). This phenomenon is likely not explained solely by the progressive decline in FFM but also by the increase in FM, which may be associated with the consequent reduction in physical activity levels with aging. This hypothesis is strengthened by the findings that the age-related decline in predicted values of REE results from not only quantitative reductions in FFM but also sedentary conditions (often associated with a qualitative reduction in functional capacity).

In these specific strata, traditional equations may offer a more comprehensive predictive potential than models strictly relying on FFM, as they implicitly account for the metabolic contribution of expanding adipose tissue. Hence, given that FFM was not the sole determinant of REE variance in our cohort—though it serves as a metabolic reference for a protective effect—clinical strategies that promote the maintenance or increase of muscle mass through exercise are key for sustaining a healthy metabolic rate regardless of sex or age group. While longitudinal studies in this specific population are needed to confirm these effects, our findings provide a clearer understanding of how body composition shifts the predictive landscape.

## Figures and Tables

**Table 1:** Equations used for estimating resting energy expenditure (REE).

Calculations		Equations
M: REE = 66.47 + (13.75 × BM) + (5.00 × height) − (6.76 × years)	(kcal/day)	(1)
F: REE = 655.10 + (9.56 × BM) + (1.85 × height) − (4.68 × years)		
M: 18–29 years − REE = (0.063 × BM + 2.896) × 239	(kcal/day)	(2)
M: 30–59 years − REE = (0.048 × BM + 3.653) × 239		
M: ≥60 − REE = (0.049 × BM + 2.459) × 239		
F: 18–29 years − REE = (0.062 × BM + 2.036) × 239		
F: 30–59 years − REE = (0.034 × BM + 3.538) × 239		
F: ≥60 − REE = (0.038 × BM + 2.755) × 239		
M: REE = (10 × BM) + (6.25 × height) − (5 × years) + 5	(kcal/day)	(3)
F: REE = (10 × BM) + (6.25 × height) − (5 × years) − 161		
REE = 19.73 × FFM + 413	(kcal/day)	(4)

Legend—M: men; F: women; REE: resting energy expenditure; BM: body mass (kg); FFM: fat-free mass; kcal: kilocalories. Height in cm; age in years. Equation (1): Harris and Benedict (1918) [11]; Equation (2): Schofield (1985) [13]; Equation (3): Mifflin et al. (1990) [12] (body mass, height, and age); Equation (4): Mifflin et al. (1990) [12] (FFM).

**Table 2:** Mean values for the characterization of the studied sample.

	Age Group (Years)					
Variables	20–29	30–39	40–49	50–59	60–69	70–79
Men						
*n*	502	146	58	40	57	44
Underweight (%)	1.8	1.4	1.7	2.5	17.5	11.4
Eutrophic (%)	61.4	49	41.4	30	33.3	43.2
Overweight (%)	32.1	41.4	36.2	47.55	49.1	45.5
Obesity (%)	8.4	8.3	20.6	20	-	-
Caucasian (%)	92	93.1	98.3	95	93	97.2
African descent (%)	8	6.9	1.7	5	7	2.35
Height (cm)	177.4 ± 7.2	178.5 ± 8.5	176.8 ± 7.2	173.4 ± 5.9	170.1 ± 6.3	168.1 ± 7.4
Weight (kg)	76.5 ± 12.0	81.0 ± 12.9	82.9 ± 12.8	81.5 ± 14.4	77.3 ± 15.9	75.4 ± 13.2
BMI (kg/m^2^)	24.7 ± 3.1	25.9 ± 3.6	27.2 ± 4.2	27.7 ± 4.5	27.3 ± 5.1	27.3 ± 4.3
FM total (kg)	14.1 ± 6.3	16.1 ± 7.5	20.5 ± 8.2	21.7 ± 7.2	22.1 ± 9.1	22.2 ± 8.2
FFM total (kg)	62.4 ± 8.9	64.9 ± 9.4	62.4 ± 7.9	59.7 ± 9.3	55.3 ± 8.7	53.2 ± 7.6
FM total (%)	18.0 ± 6.0	19.4 ± 7.0	24.1 ± 7.0	26.2 ± 5.9	27.5 ± 7.4	28.8 ± 6.6
FFM total (%)	82.0 ± 6.0	80.6 ± 7.0	75.9 ± 7.0	73.8 ± 5.9	72.5 ± 7.4	71.2 ± 6.6
Women						
*n*	268	82	92	175	325	199
Underweight (%)	4.5	2.4	3.3	2.3	5.8	8.5
Eutrophic (%)	72.8	62.2	44.6	22.9	33.2	44.7
Overweight (%)	17.9	23.2	32.6	29.1	60.9	46.7
Obesity (%)	4.8	12.1	19.6	45.7	-	-
Caucasian (%)	93.7	95.1	93.5	86.9	88	93.5
African descent (%)	6.3	4.9	6.5	13.1	12	6.5
Height (cm)	166.1 ± 8.4	164.1 ± 8.4	162.3 ± 7.6	158.0 ± 6.4	156.7 ± 6.3	154.8 ± 6.2
Weight (kg)	63.7 ± 12.2	67.2 ± 14.9	69.2 ± 13.6	73.7 ± 14.3	70.8 ± 13.8	65.5 ± 11.5
BMI (kg/m^2^)	23.5 ± 3.7	25.5 ± 5.5	26.8 ± 5.1	30.2 ± 6.1	29.5 ± 5.2	27.9 ±4.7
FM total (kg)	19.5 ± 8.2	23.0 ± 10.9	25.2 ± 8.1	30.1 ± 9.0	28.4 ± 8.5	26.4 ± 7.6
FFM total (kg)	44.1 ± 8.8	44.2 ± 8.6	44.0 ± 8.9	43.6 ± 7.3	42.4 ± 7.8	39.1 ± 6.1
FM total (%)	30.3 ± 8.5	33.3 ± 9.7	36.0 ± 7.5	40.2 ± 6.4	39.6 ± 6.3	39.8 ± 6.4
FFM total (%)	69.7 ± 8.5	66.7 ± 9.7	64.0 ± 7.5	59.8 ± 6.4	60.4 ± 6.3	60.2 ± 6.4

Legend—BMI: body mass index; FM: fat mass; FFM: fat-free mass.

**Table 3:** Comparison between FFM and FM according to physical activity level and sex in different age groups.

	Men		Women	
Variables	Active	Sedentary	Active	Sedentary
Age Group ≤ 29 years				
*n*	204	298	81	187
FFM (kg)	61.4 ± 9.3	63.1 ± 8.5 *	43.3 ± 8.6	44.5 ± 8.9
FM (kg)	12.9 ± 5.2	14.9 ± 6.8 *	19.6 ± 8.1	19.5 ± 8.4
FFM (%)	82.9 ± 5.6	81.3 ± 6.3 *	69.3 ± 8.2	69.9 ± 8.7
FM (%)	17.1 ± 5.6	18.6 ± 6.3 *	30.7 ± 8.2	30.1 ± 8.7
Age Group ≥ 30 to ≤59 years				
*n*	91	152	89	260
FFM (kg)	64.9 ± 9.5	62.5 ± 8.9	44.1 ± 9.8	43.7 ± 7.4
FM (kg)	16.9 ± 8.0	18.8 ± 7.9	26.1 ± 8.7	27.5 ± 10.1
FFM (%)	79.8 ± 8.1	77.5 ± 6.8 *	63.2 ± 7.2	62.3 ± 8.4
FM (%)	20.2 ± 8.1	22.5 ± 6.8 *	36.8 ± 7.2	37.7 ± 8.4
Age Group ≥ 60 years				
*n*	24	77	119	405
FFM (kg)	55.8 ± 8.2	53.9 ± 8.3	41.8 ± 8.0	41.0 ± 7.2
FM (kg)	23.6 ± 9.4	21.7 ± 8.4	27.8 ± 7.9	27.6 ± 8.3
FFM (%)	71.2 ± 8.1	72.2 ± 6.7	60.4 ± 6.7	60.3 ± 6.2
FM (%)	28.8 ± 8.1	27.8 ± 6.7	39.6 ± 6.7	39.7 ± 6.2

Legend—FFM: fat-free mass; FM: fat mass; * *p* ≤ 0.05 for comparisons between active and sedentary individuals within the same sex and age group.

**Table 4:** Statistical values for the comparison of REE (kcal/day) obtained from different equations.

Variables	Men	Women
Age Group ≤ 29 years		
*n*	502	268
Equation (1)	1842 ± 187 ^†‡^	1461 ± 123 ^#†‡^
Equation (2)	1843 ± 180 ^†‡^	1430 ± 180 ^¥†‡^
Equation (3)	1758 ± 149 ^¥#‡^	1396 ± 154 ^¥#‡^
Equation (4)	1642 ± 174 ^¥#†^	1282 ± 174 ^¥#†^
Age Group ≥ 30 to ≤59 years		
*n*	243	349
Equation (1)	1807 ± 211 ^†‡^	1408 ± 140 ^#†‡^
Equation (2)	1808 ± 150 ^†‡^	1422 ± 177 ^¥†‡^
Equation (3)	1731 ± 168 ^¥#‡^	1315 ± 161 ^¥#‡^
Equation (4)	1662 ± 181 ^¥#†^	1277 ± 158 ^¥#†^
Age Group ≥ 60 years		
*n*	101	524
Equation (1)	1500 ± 226	1284 ± 139 ^†‡^
Equation (2)	1483 ± 172	1283 ± 120 ^†‡^
Equation (3)	1484 ± 175	1163 ± 160 ^#†‡^
Equation (4)	1484 ± 163	1223 ± 145 ^¥†^

^¥^ difference from Equation (1); ^#^ difference from Equation (2); ^†^ difference from Equation (3); ^‡^ difference from Equation (4).

**Table 5:** Comparison between the REE estimates according to different equations, physical activity levels, sex and age groups.

	Men		Women	
Variables	Active	Sedentary	Active	Sedentary
Age Group ≤ 29 years				
*n*	204	298	81	187
Equation (1)	1806 ± 188	1866 ± 183 *	1451 ± 123	1465 ± 124
Equation (2)	1810 ± 176	1866 ± 180 *	1418 ± 176	1435 ± 182
Equation (3)	1732 ± 154	1776 ± 144 *	1388 ± 156	1399 ± 154
Equation (4)	1622 ± 182	1656 ± 168 *	1265 ± 169	1289 ± 176
Age Group ≥ 30 to ≤59 years				
*n*	91	152	89	260
Equation (1)	1829 ± 212	1795 ± 211	1403 ± 147	1410 ± 138
Equation (2)	1812 ± 141	1806 ± 156	1416 ± 124	1424 ± 116
Equation (3)	1749 ± 172	1721 ± 165	1313 ± 172	1315 ± 158
Equation (4)	1692 ± 188	1645 ± 176	1281 ± 193	1275 ± 145
Age Group ≥ 60 years				
*n*	24	77	119	405
Equation (1)	1548 ± 223	1486 ± 227	1291 ± 141	1282 ± 139
Equation (2)	1517 ± 173	1473 ± 172	1290 ± 117	1281 ± 121
Equation (3)	1523 ± 173	1473 ± 176	1169 ± 165	1161 ± 159
Equation (4)	1512 ± 162	1475 ± 163	1236 ± 157	1220 ± 142

* *p* ≤ 0.05 for comparisons between active and sedentary individuals within the same sex and age group.

**Table 6:** Spearman’s correlation between predicted values from different equations, FFM, and FM for both sexes across different age groups.

	Men		Women	
Variables	FFM	FM	FFM	FM
Age Group ≤ 29 years				
*n*	502		268	
Equation (1)	0.863 **	0.576 **	0.807 **	0.507 **
Equation (2)	0.862 **	0.616 **	0.795 **	0.557 **
Equation (3)	0.856 **	0.551 *	0.832 **	0.410 **
• Equation (4)	1.0 **	0.183 *	1.0 **	0.012
Age Group ≥ 30 and ≤ 59 years				
*n*	243		349	
Equation (1)	0.830 **	0.493 **	0.817 **	0.677 **
Equation (2)	0.785 **	0.671 **	0.776 **	0.835 **
Equation (3)	0.826 **	0.456 **	0.825 **	0.571 **
• Equation (4)	1.0 **	0.127 *	1.0 **	0.341 **
Age Group ≥ 60 years				
*n*	101		524	
Equation (1)	0.831 **	0.861 **	0.833 **	0.817 **
Equation (2)	0.978 **	0.891 **	0.828 **	0.859 **
Equation (3)	0.965 **	0.843 **	0.842 **	0.773 **
• Equation (4)	1.0 **	0.533 **	1.0 **	0.475 **

Legend—FFM: fat-free mass; FM: fat mass; * *p* < 0.05 for the comparison between equations and FFM and FM variables; ** *p* < 0.01 for the comparison between equations and FFM and FM variables. • Fixed correlation to FFM due to equation structure; used as a reference for comparison.

**Table 7:** Spearman’s correlation between predicted values from different equations, FFM, and FM according to sex and physical activity level in different age groups.

	Active				Sedentary			
	Men		Women		Men		Women	
Variables	FFM	FM	FFM	FM	FFM	FM	FFM	FM
Age Group ≤ 29 years								
*n*	204		81		298		187	
Equation (1)	0.874 **	0.592 **	0.803 **	0.566 **	0.851 **	0.567 **	0.810 **	0.487 **
Equation (2)	0.883 **	0.612 **	0.815 **	0.590 **	0.843 **	0.616 **	0.791 **	0.542 **
Equation (3)	0.866 **	0.569 **	0.823 **	0.471 **	0.842 **	0.538 **	0.837 **	0.383 **
• Equation (4)	1.0 **	0.214 **	1.0 **	0.093	1.0 **	0.156 **	1.0 **	−0.011
Age Group ≥ 30 to ≤59 years								
*n*	91		89		152		260	
Equation (1)	0.783 **	0.437 **	0.891 **	0.572 **	0.857 **	0.556 **	0.787 **	0.710 **
Equation (2)	0.729 **	0.638 **	0.830 **	0.785 **	0.825 **	0.715 **	0.750 **	0.851 **
Equation (3)	0.787 **	0.375 **	0.907 **	0.452 **	0.852 **	0.528 **	0.795 **	0.608 **
• Equation (4)	1.0 **	0.002	1.0 **	0.370 **	1.0 **	0.249 *	1.0 **	0.324 **
Age Group ≥ 60 years								
*n*	24		119		77		405	
Equation (1)	0.719 **	0.870 **	0.818 **	0.746 **	0.858 **	0.862 **	0.840 **	0.835 **
Equation (2)	0.750 **	0.887 **	0.812 **	0.775 **	0.862 **	0.891 **	0.834 **	0.879 **
Equation (3)	0.723 **	0.851 **	0.836 **	0.701 **	0.861 **	0.842 **	0.846 **	0.792 **
• Equation (4)	1.0 **	0.428 *	1.0 **	0.345 **	1.0 **	0.572 **	1.0 **	0.513 **

Legend—FM: fat mass; FFM: fat-free mass; * *p* ≤ 0.05 and ** *p* ≤ 0.01 for comparisons between active and sedentary individuals within the same sex. • Fixed correlation to FFM due to equation structure; used as a reference for comparison.

**Table 8:** Linear regression of different equations on FFM and FM for both sexes.

	Men						Women					
	FFM			FM			FFM			FM		
	R^2^	SEE (kcal)	β	R^2^	SEE (kcal)	β	R^2^	SEE (kcal)	β	R^2^	SEE (kcal)	β
Equation (1)	0.737	114.2	20.7	0.148	202.2	11.0	0.620	92.2	14.9	0.251	132.2	8.2
Equation (2)	0.685	112.5	17.9	0.131	186.1	9.5	0.625	86.7	14.2	0.331	113.7	8.8
Equation (3)	0.734	91.3	16.4	0.127	162.8	8.1	0.636	109.0	18.3	0.167	166.1	7.9

Legend—FFM: fat-free mass; FM: fat mass; SEE: standard error of estimate; β: beta coefficient. Sample size differed for each equation following the criteria for eliminating residual outliers, as follows: Equation (1) FFM (men = 839, women = 1131—excluded cases: seven (0.8%) and nine (0.8%), respectively); Equation (1) FM (men = 839, women = 1133—excluded cases: seven (0.8%) and eight (0.7%), respectively); Equation (2) FFM (men = 841, women = 1122—excluded cases: five (0.6%) and nineteen (1.7%), respectively); Equation (2) FM (men = 842, women = 1120—excluded cases: four (0.5%) and twenty-one (1.9%), respectively); Equation (3) FFM (men = 838, women = 1132—excluded cases: eight (0.9%) and nine (0.8%), respectively); Equation (3) FM (men = 839, women = 1132—excluded cases: seven (0.8%) and nine (0.8%), respectively). Regression ANOVA showed *p* < 0.01 for FFM and FM in all equations for both sexes.

## Data Availability

Data supporting this study’s findings are available from the corresponding author upon reasonable request.

## Abbreviations

The following abbreviations are used in this manuscript:

REE	Resting Energy Expenditure
BMR	Basal Metabolic Rate
FFM	Fat-Free Mass
FM	Fat Mass
DXA	Dual-Energy X-ray Absorptiometry
IC	Indirect Calorimetry
BMI	Body Mass Index
SEE	Standard Error of Estimate
β	Beta Coefficient (statistical regression)
